# Supplementary material for: Identification of Novel Src Inhibitors: Pharmacophore-Based Virtual Screening, Molecular Docking and Molecular Dynamics Simulations
Source: Molecules. 2020 Sep 8;25(18):4094. doi: 10.3390/molecules25184094 (PMC7571137; doi:10.3390/molecules25184094)
Supplement: Supplementary file 1 [file molecules-25-04094-s001.pdf]

molecules

MDPI

Supplementary Materials

Identification of Novel Src Inhibitors: Pharmacophore Based Virtual Screening, Molecular Docking and Molecular Dynamics Simulations

Yi Zhang, Ting-jian Zhang, Shun Tu, Zhen-hao Zhang and Fan-hao Meng*

School of Pharmacy, China Medical University, Shenyang 110122, China; cruckzhang0304@163.com (Y.Z.); todayfy@outlook.com (T.-j.Z.); tushun2018@163.com (S.T.); zhangzhenhao314@163.com (Z.-h.Z.)

* Correspondence: fhmeng@cmu.edu.cn; Tel.: +86-133-8688-7639

ADME

ID	Value
BBB	0.0103644**
Buffer_solubility_mg_L	81.6862**
Caco2	18.8714
CYP_2C19_inhibition	Non
CYP_2C9_inhibition	Non
CYP_2D6_inhibition	Non
CYP_2D6_substrate	Non
CYP_3A4_inhibition	Inhibitor
CYP_3A4_substrate	Substrate
HIA	97.409918
MDCK	0.0440467**
Pgp_inhibition	Inhibitor
Plasma_Protein_Binding	100.000000**
Pure_water_solubility_mg_L	0.000456487**
Skin_Permeability	-1.95856**
SKlogD_value	5.861190**
SKlogP_value	5.861190**
SKlogS_buffer	-3.796760**
SKlogS_pure	-9.049480**

Toxicity

ID	Value
algae_at	0.00280111**
Ames_test	mutagen
Carcino_Mouse	negative
Carcino_Rat	negative
daphnia_at	0.00222569**
hERG_inhibition	low_risk
medaka_at	1.91433e-005**
minnow_at	4.47499e-005**
TA100_10RLI	negative
TA100_NA	negative
TA1535_10RLI	negative
TA1535_NA	negative

Figure S1. The ADMET prediction for ZINC3214460

ADME

ID	Value
BBB	0.449141
Buffer_solubility_mg_L	3735.39**
Caco2	26.6213
CYP_2C19_inhibition	Non
CYP_2C9_inhibition	Non
CYP_2D6_inhibition	Non
CYP_2D6_substrate	Substrate
CYP_3A4_inhibition	Non
CYP_3A4_substrate	Substrate
HIA	92.105528
MDCK	2.5236
Pgp_inhibition	Non
Plasma_Protein_Binding	34.897436
Pure_water_solubility_mg_L	432.56
Skin_Permeability	-4.56456
SKlogD_value	1.283610
SKlogP_value	1.283610
SKlogS_buffer	-2.026970**
SKlogS_pure	-2.963260

Toxicity

ID	Value
algae_at	0.0608198
Ames_test	mutagen
Carcino_Mouse	negative
Carcino_Rat	negative
daphnia_at	0.15396
hERG_inhibition	high_risk
medaka_at	0.0490291
minnow_at	0.250973
TA100_10RLI	negative
TA100_NA	positive
TA1535_10RLI	negative
TA1535_NA	negative

Figure S2. The ADMET prediction for ZINC1380384
